# Assessing the need for general anesthesia versus inhalational sedation using modified mohan scale in pediatric dental patients

**DOI:** 10.1016/j.jobcr.2025.08.015

**Published:** 2025-08-19

**Authors:** Sriram Swetha, Bagmar R. Mehul, Jayakumar Priya

**Affiliations:** Department of Pediatric and Preventive Dentistry, Sri Ramachandra Dental College and Hospital, Sri Ramachandra Institute of Higher Education and Research, Porur, Chennai, 600 116, Tamil Nadu, India

**Keywords:** General anesthesia, Inhalational sedation, Behavior management, Dental anxiety, Nitrous Oxide Sedation

## Abstract

**Background:**

Effective behavior management is vital in pediatric dentistry to ensure positive treatment in uncooperative children. General anesthesia (GA) and Inhalational sedation (IS) are some commonly used techniques, but an objective assessment tool is required to determine the most appropriate approach.

**Objective:**

This study aims to assess the effectiveness of the Modified Mohan Scale in guiding the selection between GA and IS for pediatric dental patients.

**Methods:**

Thirty children aged 3–6 years were evaluated using the Modified Mohan Scale, which considers American Society of Anesthesiologists (ASA) classification, treatment complexity, number of teeth affected, behavior during radiographic imaging, parental expectations, socioeconomic status, and Frankl's Behavior Rating Scale. Mann–Whitney *U* test was conducted after assessing normality with the Shapiro–Wilk test to compare the GA and IS groups.

**Results:**

Significant differences were found in the total Modified Mohan Scale scores between the GA and IS groups (p = 0.0001). Additionally, subscales related to the number of teeth requiring treatment (p = 0.011) and behavior during radiographic imaging (p = 0.029) showed prominent variations, with higher scores indicating a greater need for GA.

**Conclusion:**

The Modified Mohan Scale shows promising potential as a structured and consistent method for evaluating the need for GA versus IS in pediatric dental patients. The scale's application may support clinical decision-making and treatment planning. Future research with a larger sample size is recommended to validate its application for broader use.

## 1 Introduction

Pharmacological behavior management techniques are often employed in Pediatric Dentistry to handle children lacking cooperative ability. General anesthesia (GA) and Inhalational Sedation (IS) techniques are two commonly utilized methods for managing behavior, especially in anxious children or those with special healthcare requirements. These interventions significantly reduce anxiety, and enable safe, efficient treatment.^1^ Additionally, pharmacological aids minimize pain perception, thereby enhancing patient comfort and cooperation during treatments.^1^ The choice between GA and IS depends on several factors such as parental preference, access to facilities, quantity of treatment, cost, practitioner preference, and most importantly, the child's behavior.^2^, ^3^, ^4^

A study by Eidelman E et al., reported that restorative treatment performed under GA yielded better results than those under sedation. However, treatment success was more prevalent in patients treated under sedation who exhibited “positive” behavior (Marginal Adaptation - 96 %; Anatomic Form - 96 %; Secondary Caries Absence - 94 %).^5^ Additionally, a study by Shaw AJ et al., found that extractions performed under sedation were clinically successful, more acceptable and cost-effective.^6^ Nevertheless, due to the limitations of subjective decision alone, the necessity for structured, objective tools in clinical decision-making has gained increasing consideration. The Indicator of Sedation Need (IOSN) tool is used in adults to evaluate the necessity of dental sedation by considering self-reported anxiety ratings and prevalent medical problems.^7^ However, it relies on self-reported anxiety levels, adult health conditions, and procedural complexity. While the IOSN tool is generally used in adult sedation assessment, it does not completely account for pediatric considerations such as developmental stage and behavior variability. Thus, it is unsuitable for assessing pediatric patients or guiding GA versus IS decisions.^2^

It is imperative to develop a systematic, objective scale to support sedation decision-making. Such a scale would enable the standardization of care protocols for pediatric patients. This study builds on the foundation of the original Mohan scale, which was designed to assess the need for GA or conscious sedation in pediatric dental patients.^2^ The original Mohan Scale encompassed seven subscales focusing principally on medical status, age, weight, extent and complexity of treatment, and cooperation levels, and was validated in a hospital setting in the United States. In this study, we expanded the scale to ten subscales by integrating additional factors to improve its appropriate relevance and clinical efficacy for the pediatric dental population in India. The objective of this study is to evaluate the necessity of GA versus IS in Indian pediatric patients lacking co-operative ability by modifying the original Mohan scale. These modifications aim to improve clinical relevance and provide a structured tool for decision-making. The use of a standardized and objective decision-making tool is clinically significant as it aids in ensuring reliability in care, reduces practitioner bias, and enhances patient outcomes. Such tools can also support active communication with caregivers and improve overall treatment planning.

## 2 Methods

### Ethical approval

This study was carried out after obtaining approval from the Institutional Ethical Committee (IEC) – CSP/23/APR/126/292. A written informed consent was collected from each participant's primary caregiver before the study commenced.

## 3 Participants selection

### 3.1 Inclusion criteria

The inclusion criteria consisted of children: (i) between 3 and 6 years of age who have not undergone any previous dental management, (ii) all genders, and (iii) who were healthy.

### 3.2 Exclusion criteria

The exclusion criteria included children: (i) who have undergone previous dental treatment, (ii) whose parents were not willing to participate in the study, and (iii) with special health care needs.

### 3.3 Sample size

Based on a mean difference of 8 in total scores between the IS and GA groups, with standard deviations of 5 and 7, respectively, the sample size was calculated with 90 % statistical power and a 5 % alpha error. The estimated sample size was 14 participants per group, which was then adjusted to 15 per group, resulting in a total sample size of 30 participants. The sample size estimation for a two-sample means test was conducted using Satterthwaite's *t*-test, assuming unequal variances.

### 3.4 Modified Mohan’s scale

**Fig. 1:**
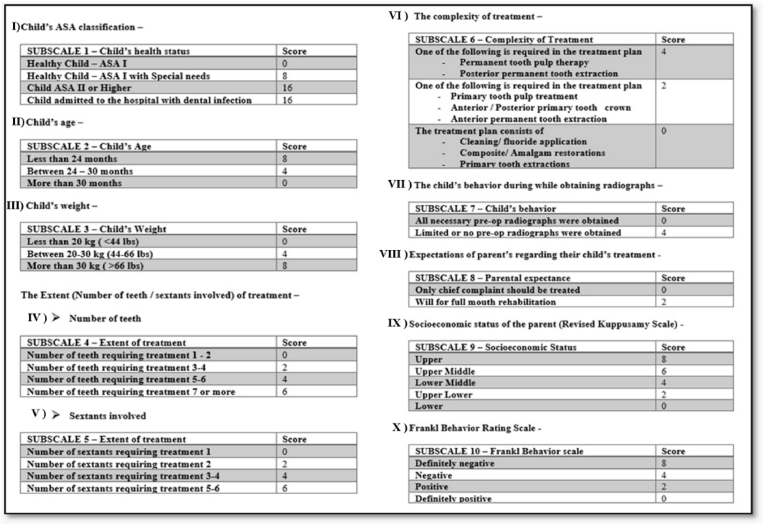
Modified criteria to assess the need for General anesthesia versus inhalation sedation.

The Mohan scale initially comprised seven subscales, each assigned a designated score, with the total score being the aggregate of all subscale scores. This scale was utilized to assess the need for GA or IS in pediatric dental patients. Higher overall scores suggested a greater likelihood of requiring GA over IS.^2^ In the present study, a modified version of Mohan's scale was utilized, which included ten subscales, with each subscale having a defined scoring range. Permission to use and modify the scale was obtained from the original author via email. Each subscale was scored independently, and the total score was the sum of all ten. The subscales included the child's American Society of Anesthesiologists (ASA) classification, age, weight, extent (number of teeth/sextants involved) of treatment, complexity of treatment, behavior while obtaining radiographs, parental expectance, socioeconomic status of the parent^8^ and the behavior according to the Frankl's rating scale (Fig. 1). Based on expert clinical consensus among the investigators and due to the addition of new subscales, which expanded the total score range, a cutoff score of 25 was applied to distinguish between cases suitable for GA versus IS. Children scoring 25 or higher were considered candidates for GA.

## 4 Study design

The study included children aged 3–6 years who were chosen from the outpatient department of Pediatric and Preventive Dentistry at Sri Ramachandra Dental College and Hospital. The Modified Mohan Scale was applied by a pediatric dental postgraduate student (SS) and a dental undergraduate intern (MB). Before data collection, the investigators underwent calibration sessions under the supervision of a senior pediatric dentist (PJ) to ensure consistency in scoring. Participants were recruited using a consecutive sampling method as they presented at the department and met the inclusion criteria during the data collection period in June 2023. During their initial dental visit, the investigators filled each scale and collected information regarding the child's ASA classification, age, weight, extent (number of teeth/sextants involved) of treatment, complexity of treatment, behavior while obtaining radiographs, parental expectance, socioeconomic status of the parent^8^ obtained from the accompanying parent or guardian during clinical interview using the Revised Kuppuswamy Scale (2017) and the behavior according to the Frankl's rating scale. The data was entered in a data extraction form, which was then entered on a Microsoft Excel sheet.

## 5 Statistical analysis

The analysis involved both descriptive and inferential statistics, performed using IBM SPSS version 20.0 (IBM Corp. Released 2011. IBM SPSS Statistics for Windows, Version 20.0. Armonk, NY: IBM Corp). Quantitative data (Age, Modified Mohan's score, and other variables) were summarized using mean and standard deviation (SD). Frequency and Percentage were used to summarize gender distribution. Data normality was assessed using the Shapiro-Wilk test (Table 1). Based on the results, the non-parametric Mann–Whitney *U* test was used to compare quantitative variables between the two groups. A P-value of less than 0.05 was regarded as statistically significant throughout the study.

**Table 1:** depicts the Shapiro-Wilk Normality test.

Group		Shapiro-Wilk		
		Statistic	df	P value
**Inhalational Sedation**	**Number of teeth**	**0.861**	**15**	**0.025**
	**Sextants involved**	**0.805**	**15**	**0.004**
	**Complexity of treatment**	**.**	**15**	**.**
	**Behavior while imaging**	**0.284**	**15**	**<0.001**
	**Expectations of parent's regarding their child's treatment**	**0.499**	**15**	**<0.001**
	**Socioeconomic status of the parent**	**0.799**	**15**	**0.004**
	**Frankl Behavior Rating Scale**	**0.561**	**15**	**<0.001**
	**Modified Mohan's Score**	**0.920**	**15**	**0.191**
**General Anesthesia**	**Number of teeth**	**0.744**	**15**	**<0.001**
	**Sextants involved**	**0.714**	**15**	**<0.001**
	**Complexity of treatment**	**.**	**15**	**.**
	**Behavior while imaging**	**0.643**	**15**	**<0.001**
	**Expectations of parent's regarding their child's treatment**	**.**	**15**	**.**
	**Socioeconomic status of the parent**	**0.766**	**15**	**0.001**
	**Frankl Behavior Rating Scale**	**0.730**	**15**	**<0.001**
	**Modified Mohan's Score**	**0.763**	**15**	**0.001**

## 6 Results

**Fig. 2:**
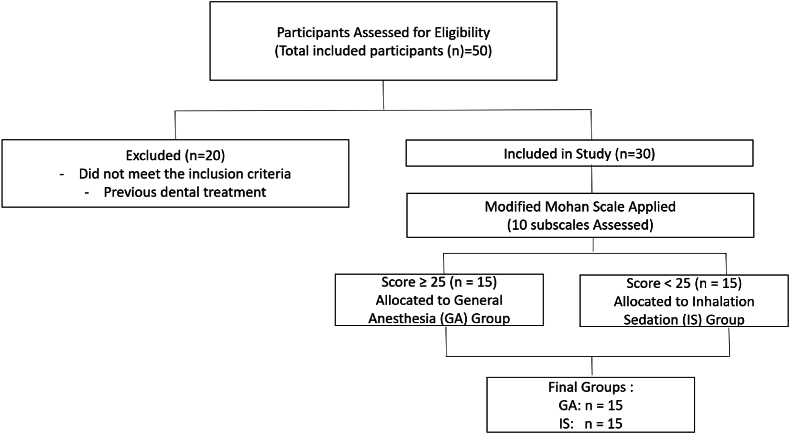
Flow diagram showing participant screening and final group allocation into General Anesthesia (GA) and Inhalational Sedation (IS) groups.

**Fig. 3:**
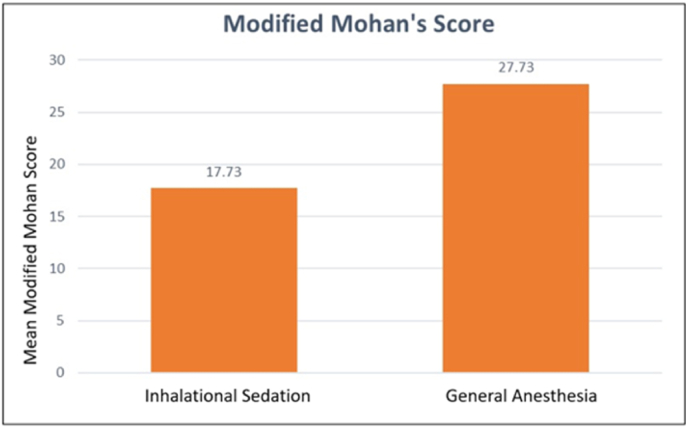
Bar chart comparing the mean Modified Mohan Scale scores between the two groups.

The study involved 30 children aged 3–6 years. Based on the scores obtained, 15 children were identified as requiring GA, while the other 15 were assigned to the IS group (Fig. 2). None of the participants had previously received any dental interventions. The mean age of the children in the IS group was 4.66 years, compared to 3.73 years in the GA group. Table 2 illustrates the participants' mean age and gender distribution. Intergroup comparisons, conducted using the Mann-Whitney *U* test, revealed a significant difference in the Modified Mohan's total score between the groups (p-value = 0.0001), as well as significant differences in the number of teeth subscale (p-value = 0.011) and the behavior while obtaining radiograph subscale (p-value = 0.029) (Table 3). A bar chart comparing the mean Modified Mohan Scale scores is depicted in Fig. 3.

**Table 2:** depicts the descriptive statistics.

Group	Mean age	Standard Deviation
*Inhalational*	4.66	0.487
*General Anesthesia*	3.73	0.942
**Gender distribution**	**Males (in percentage)**	**Female (in percentage)**
*Inhalational*	40	60
*General Anesthesia*	33.3	66.7

**Table 3:** depicts the Mann-Whitney *U* test.

Variables in Scale	Group	Mean ± Standard Deviation	Mean Rank	Sum of Ranks	Z	P-value
Number of teeth	Inhalational	3.20 ± 1.82	11.50	172.50	2.630	**0.011**
	General Anesthesia	4.93 ± 1.28	19.50	292.50		
Sextants involved	Inhalational	3.20 ± 1.66	12.40	186.00	2.191	0.056
	General Anesthesia	4.40 ± 1.55	18.60	279.00		
Complexity of treatment	Inhalational	2.00 ± 0.00	15.50	232.50	0.00	∗
	General Anesthesia	2.00 ± 0.00	15.50	232.50		
Behavior while imaging	Inhalational	0.27 ± 1.03	12.00	180.00	2.742	**0.029**
	General Anesthesia	2.13 ± 2.07	19.00	285.00		
Expectations of parent's regarding their child's treatment	Inhalational	1.60 ± 0.83	14.00	210.00	1.795	0.367
	General Anesthesia	2.00 ± 0.00	17.00	255.00		
Socioeconomic status of the parent	Inhalational	4.00 ± 1.31	13.50	202.50	1.4	0.217
	General Anesthesia	4.67 ± 1.23	17.50	262.50		
Frankl Behavior Rating Scale	Inhalational	2.53 ± 0.92	13.23	198.50	1.631	0.161
	General Anesthesia	3.60 ± 2.03	17.77	266.50		
Modified Mohan's Score	Inhalational	17.73 ± 3.92	8.00	120.00	4.274	**0.0001**
	General Anesthesia	27.73 ± 2.25	23.00	345.00

## 7 Discussion

Children's fear of dental treatment poses significant challenges for families and the dental profession, particularly for pediatric dentists.^9^ Numerous factors influence children's dental anxiety and fear, such as maternal stress, knowledge of dental issues, prior experiences, and unfamiliar sounds or smells.^10^ Even mild levels of anxiety can result in inconsistent appointments and insufficient follow-up care. Intense anxiety can lead to disrupted sleep, negative thought patterns, reduced self-esteem, and depression.^11^ Managing dental anxiety can involve behavioral control strategies, the administration of sedatives and hypnotics, or a combination of both.^1^ Each of these strategies has its own benefits and limitations. The use of pharmacological interventions is essential for managing dental anxiety. However, the selection of appropriate pharmacological agents depends on various factors.

A child's temperament and behavior can significantly influence the long-term effectiveness of IS.^3^ Many other considerations, such as practitioner preference^12^^,^^13^, parental preference^14^^,^^15^, availability to facilities, safety,^4^^,^^5^ and treatment cost, may affect the choice to utilize GA over IS. The patient's age also plays a role in determining whether GA or IS is appropriate.^16^ Considering these factors, the majority of the children cannot be treated completely without pharmacological behavior management. Moreover, when used appropriately, these pharmacological tools enhance the overall dental experience, strengthening patient-dentist relationships and contributing to better long-term oral health outcomes.^1^

In this study, a set of pre-existing subscales^2^ was modified to assess their effectiveness in determining the use of IS versus GA for providing dental care to uncooperative children. The developed subscales include the status of the child's health, age, weight, extent and complexity of treatment, behavior, parental expectations, socioeconomic status, and the Frankl behavior scale. Generally, a healthy child will score better on the subscale compared to a child prone to infections. Older children are typically more cooperative than younger children. These criteria were designed to assist clinicians create appropriate treatment plans for uncooperative pediatric patients.

A 2008 survey revealed that physicians' clinical care decisions were influenced by the socioeconomic status and educational qualifications of their patients. Therefore, a parent's socioeconomic background and education level can significantly impact the treatment course and outcome of medical and dental care. This influence also extends to treatments under conscious sedation, where children avoid unpleasant experiences. Gharavi and Soltani reported that dentists frequently recommend GA for patients with disabilities or those who are uncooperative, as it ensures favorable outcomes with minimal complications.^17^ Nunn and colleagues emphasized the need for extensive treatment in children with medical issues, disabilities, severe anxiety, younger age, or those residing far from dental facilities, making GA a definitive solution.^18^ Furthermore, parental expectations and socioeconomic status (using the Kuppuswamy scale) play a crucial role.^8^ Parents who desire comprehensive treatment and belong to higher socioeconomic classes typically achieve higher scores on the subscale. One advantage of therapy under GA, as shown by Reich et al., is the absence of unpleasant recollections after dental treatments.^19^

Consequently, the last criterion in the subscale is the Frankl behavior rating scale, where a child with definitely negative behavior is scored higher than one with definitely positive behavior. Heidari et al., in 2022 identified key reasons for using GA in pediatric dental procedures. These included younger age, poor cooperation during past treatments, extensive treatment requirements, and minimizing the number of sessions.^20^ In the present scale, the extent of treatment is divided into the number of teeth to be treated and the number of sextants involved. Thus, a higher number of teeth and sextants results in a higher score on the subscale. Treatment complexity is also evaluated, with simple procedures like scaling and fluoride varnish receiving lower scores compared to complex treatments like pulp therapy and extractions. Additionally, child behavior during radiograph procedures is also considered. Pohl and colleagues found that lack of cooperation in dental treatments often necessitates GA.^21^ Consequently, a cooperative child, who allows all necessary treatments and preoperative radiographs, will score better than an uncooperative child according to the scoring criteria.

The Modified Mohan Scale represents a novel and structured approach specifically tailored for pediatric dental care, addressing a significant clinical gap in sedation planning. Its comprehensive framework enhances decision-making by combining medical, behavioral, procedural, and socioeconomic factors that are particularly relevant in Indian pediatric practice. A major strength of this study is its novel methodology for assessing the necessity of GA versus IS in pediatric patients within the Indian population using a modified assessment scale, addressing a gap previously unexamined in the literature. The study utilizes a comprehensive, multi-dimensional assessment tool that considers various factors, including the status of the child's health, age, weight, treatment complexity, behavior, parental expectations, and socioeconomic status. While the original Mohan Scale used a cutoff of 18 determined by ROC analysis, the present study introduced three additional subscales, which altered the score distribution. Accordingly, a new cutoff of 25 was selected based on investigator consensus. However, this threshold was not statistically validated, and future studies with larger samples should confirm its diagnostic performance through formal validation techniques such as ROC analysis. Additionally, the limited sample size and single-center design reduce the generalizability of the findings. The exclusion of children with previous dental treatments and special health care needs may further restrict the relevance of the study's findings to all pediatric dental patients. The absence of long-term treatment outcome assessment and potential subjectivity in scoring some subscales also represent limitations. Furthermore, the use of a hospital-based sample may introduce selection bias. Formal validation and reliability testing of the modified scale in broader settings are recommended.

Future research should focus on incorporating a larger, more diverse sample across multiple institutions to enhance the robustness and generalizability of the findings. Longitudinal studies examining long-term outcomes of treatments performed under GA versus IS would also offer valuable insights into their efficacy and safety.

## Conclusion

The present study allows for the following conclusions to be made:

1. Modified Mohan's Scale shows promising potential as a structured tool to assist clinical decision-making between GA and IS in uncooperative pediatric dental patients. However, further validation in a larger and more diverse population is warranted.
2. Parental involvement may influence treatment planning and outcomes, and therefore, future studies should explore this relationship.
3 Close collaboration between practitioner expertise and parental input is recommended to support effective management strategies for uncooperative pediatric patients.

## Ethics statement

Written consent was obtained from all participants. The Institutional Review Board of Sri Ramachandra Institution of Higher Education and Research approved the study design, data-collection methods, and the procedure for obtaining informed consent (REF: CSP/23/APR/126/292).

## Source(s) of funding

No funding was secured for this study.

## Declaration of competing interest

The authors declare that they have no known competing financial interests or personal relationships that could have appeared to influence the work reported in this paper.
